# Supplementary material for: Complexome profiling of the Chlamydomonas psb28 mutant reveals TEF5 as an early PSII assembly factor
Source: Plant Cell. 2025 Mar 17;37(6):koaf055. doi: 10.1093/plcell/koaf055 (PMC12164590; doi:10.1093/plcell/koaf055)
Supplement: koaf055_Supplementary_Data [file koaf055_supplementary_data.zip › psb28_interactive.zip!/plots/intensity/qualityPlots/peptides/_Cre06.g251850.t1.2.html]

### Cre06.g251850.t1.2

Trivial name:   
SeqCoverage [median]: 25.40  
Mass: 7.16 kDa  
  
 

| Sequence | MissedCleavage | Mass [Da] | wtIntensity | muIntensity | Score |
| --- | --- | --- | --- | --- | --- |
| DVLGEGTLNQLR | 0 | 1313.69 | 2371.32 | 8858.33 | 161.17 |
| DVLGEGTLNQLRR | 1 | 1469.80 | 4756.65 | 8329.51 | 53.97 |
| MTAKDVLGEGTLNQLRR | 2 | 1901.02 | 586.02 | 0.00 | 41.97 |
|  |  |  | 7714.0 | 17187.8 |  |
